# Cost-effectiveness of different strategies for diagnosis of uncomplicated urinary tract infections in women presenting in primary care

**DOI:** 10.1371/journal.pone.0188818

**Published:** 2017-11-29

**Authors:** Judith E. Bosmans, Veerle M. H. Coupé, Bart J. Knottnerus, Suzanne E. Geerlings, Eric P. Moll van Charante, Gerben ter Riet

**Affiliations:** 1 Department of Health Sciences, Faculty of Science, Vrije Universiteit Amsterdam, Amsterdam Public Health research institute, Amsterdam, The Netherlands; 2 Department of Epidemiology and Biostatistics, VU University Medical Centre, Amsterdam, the Netherlands; 3 Department of General Practice, Academic Medical Center, Amsterdam, the Netherlands; 4 Department of Internal Medicine / Infectious Diseases, Academic Medical Center, Amsterdam, The Netherlands; TNO, NETHERLANDS

## Abstract

**Background:**

Uncomplicated Urinary Tract Infections (UTIs) are common in primary care resulting in substantial costs. Since antimicrobial resistance against antibiotics for UTIs is rising, accurate diagnosis is needed in settings with low rates of multidrug-resistant bacteria.

**Objective:**

To compare the cost-effectiveness of different strategies to diagnose UTIs in women who contacted their general practitioner (GP) with painful and/or frequent micturition between 2006 and 2008 in and around Amsterdam, The Netherlands.

**Methods:**

This is a model-based cost-effectiveness analysis using data from 196 women who underwent four tests: history, urine stick, sediment, dipslide, and the gold standard, a urine culture. Decision trees were constructed reflecting 15 diagnostic strategies comprising different parallel and sequential combinations of the four tests. Using the decision trees, for each strategy the costs and the proportion of women with a correct positive or negative diagnosis were estimated. Probabilistic sensitivity analysis was used to estimate uncertainty surrounding costs and effects. Uncertainty was presented using cost-effectiveness planes and acceptability curves.

**Results:**

Most sequential testing strategies resulted in higher proportions of correctly classified women and lower costs than parallel testing strategies. For different willingness to pay thresholds, the most cost-effective strategies were: 1) performing a dipstick after a positive history for thresholds below €10 per additional correctly classified patient, 2) performing both a history and dipstick for thresholds between €10 and €17 per additional correctly classified patient, 3) performing a dipstick if history was negative, followed by a sediment if the dipstick was negative for thresholds between €17 and €118 per additional correctly classified patient, 4) performing a dipstick if history was negative, followed by a dipslide if the dipstick was negative for thresholds above €118 per additional correctly classified patient.

**Conclusion:**

Depending on decision makers’ willingness to pay for one additional correctly classified woman, the strategy consisting of performing a history and dipstick simultaneously (ceiling ratios between €10 and €17) or performing a sediment if history and subsequent dipstick are negative (ceiling ratios between €17 and €118) are the most cost-effective strategies to diagnose a UTI.

## Introduction

Uncomplicated urinary tract infections (UTIs) are infections of the lower urinary tract in otherwise healthy, non-pregnant, adult women without known anatomical or functional abnormalities of the urinary tract. Sixty percent of all women experience at least one UTI during their life.[1] The main symptoms are painful and frequent micturition. Considering the high incidence of these symptoms, the economic consequences of UTIs in primary care are substantial.[1]

Since antimicrobial resistance against antibiotics used for UTIs is rising [2,3], accurate diagnosis is needed to target the use of antibiotics in settings with low rates of multidrug-resistant bacteria. The diagnosis of UTI is made based on the presence of UTI symptoms in combination with bacteriuria.[4,5,6,7] Although, ideally, a urine culture should be performed to assess bacteriuria, this would result in high costs and a diagnostic delay of several days. Instead, in practice, history taking, urine stick tests, microscopic examination of the urinary sediment and a dipslide (= semi-quantitative culture) are used to diagnose a UTI.[8,9,10,11] Different combinations of history questions and urine investigations are used in clinical practice, representing different diagnostic strategies. However, it is not clear which strategy should be used to achieve high diagnostic accuracy at minimal (material and labour) costs. Therefore, we compared the cost-effectiveness of different strategies to diagnose UTIs in women who contact their general practitioner (GP) with painful and/or frequent micturition.

## Materials and methods

### Design of the study

Decision trees were used to compare the costs and effects of the different strategies to diagnose UTIs in women who contact their general practitioner (GP) with painful and/or frequent micturition that had been present for no longer than seven days.

### Data

Data on the diagnostic accuracy of the tests included in the diagnostic strategies were obtained from a previously published diagnostic cohort study conducted in and around Amsterdam, The Netherlands.[12] In this study, women who contacted their GP between April 2006 and October 2008 with painful and/or frequent micturition underwent the four tests described below as well as a urine culture (which was performed and assessed independently from the other four tests). The urine culture was considered the gold standard for diagnosing a UTI. A UTI was considered present if there were more than 10^3^ colony-forming units (CFUs) of a single uropathogen per milliliter (mL) of urine [13].

Four different diagnostic tests were performed in the observational study:

- History (positive if a woman suspected a UTI and reported at least considerable pain during micturition (score 3 or 4 on a 1–4 scale))
- Dipstick (positive if nitrite was detected)
- Sediment, performed in a laboratory by trained laboratory technicians (positive if there were >20 leucocytes/ high-power field (HPF))
- Dipslide (positive if there were ≥10^5^ colony-forming units (CFU) of a single uropathogen per milliliter (mL) on cystine lactose electrolyte deficient (CLED) medium)

### Diagnostic strategies

Diagnostic strategies were based on clinical guidelines and current practice, and varied with respect to the combination and order of tests. Using different combinations and sequences of these four tests, 15 strategies were defined based on clinical guidelines and current practice. Details of each strategy are shown in Table 1 and S1 Appendix. Briefly, strategies 1, 6 and 11 consist of a single test, strategies 2–5 and 13–15 comprise sequential testing (additional testing conditional on the result of the previous test), and strategies 7–10 and 12 represent parallel testing (more than one test performed simultaneously). All strategies were terminated if there was a positive test result, with the exception of strategy 13 that continued with a stick if the history result was positive. The parallel testing strategies 7, 8, 9, 10 and 12 were based on multivariable regression models comprising the individual components of the included tests; the overall result was considered positive if the predicted UTI risk was >70%.[12] For each strategy, a decision tree was constructed (S1 Appendix).

**Table 1:** Diagnostic strategies evaluated in the decision trees derived from empirical data on 196 women contacting their GP with painful and/or frequent micturition.

Strategy	1st test (TP/FP/TN/FN)	2nd test (TP/FP/TN/FN)	3rd test (TP/FP/TN/FN)	4th test (TP/FP/TN/FN)	Overall accuracy			
					PPV	NPV	Sens	Spec
1	history (66/15/61/54)				0.81	0.53	0.55	0.80
2	history, if negative (66/15/61/54)	dipstick (29/1/60/25)			0.86	0.71	0.79	0.79
3	history, if negative (66/15/61/54)	dipstick, if negative (29/1/60/25)	sediment (10/3/57/15)		0.85	0.79	0.88	0.75
4	history, if negative (66/15/61/54)	dipstick, if negative (29/1/60/25)	dipslide (9/1/60/15)		0.86	0.79	0.87	0.78
5	history, if negative (66/15/61/54)	dipstick, if negative (29/1/60/25)	sediment, if negative (10/3/57/15)	dipslide (3/1/57/11)	0.84	0.84	0.91	0.74
6	dipstick (60/3/73/60)				0.95	0.55	0.50	0.96
7	history & dipstick (72/4/72/48)				0.95	0.60	0.60	0.95
8	history & dipstick & sediment (91/9/67/29)				0.91	0.70	0.76	0.88
9	history & dipstick & dipslide (86/3/73/34)				0.97	0.68	0.72	0.96
10	history & dipstick & sediment & dipslide (91/4/72/29)				0.96	0.71	0.76	0.95
11a	dipslide ≥10^3^ CFU/mL (100/33/43/20)				0.75	0.68	0.83	0.57
11b	dipslide ≥10^5^ CFU/mL (84/7/69/36)				0.92	0.66	0.70	0.91
12	dipstick & dipslide (87/3/73/33)				0.97	0.69	0.73	0.96
13	history, if positive (66/15/61/54)	dipstick (46/35) (42/4/12/23)			0.91	0.48	0.35	0.95
14	dipstick, if negative (60/3/73/60)	history (45/88) (31/14/58/30)			0.83	0.66	0.75	0.76
15	dipstick, if negative (60/3/73/60)	dipslide (31/102) (28/3/70/32)			0.94	0.69	0.73	0.92

TP = true positives; FP = false positives; TN = true negatives; FN = false negatives; PPV = positive predictive value; NPV = negative predictive value; Sens = sensitivity; Spec = specificity

In the last four columns, positive and negative predictive values as well as conditional sensitivities and specificities for each complete strategy are shown. In particular, a conditional sensitivity means that, e.g. in a two-test strategy with two binary tests, the sensitivity of test 2 is calculated separately for those with a positive and negative result on test 1, respectively. For the dipslide, a cut-off value of ≥10^5^ CFU/mL was used in all strategies, except for strategy 11a. This strategy consisted of the dipslide as a single test at a cut-off value of ≥10^3^ CFU/mL.

### Outcome measures

#### Correctly classified women

The primary outcome used in the analysis was the percentage of women correctly classified either with or without a UTI. For each strategy, the proportion of correctly classified patients was estimated by summing the number of true-positive and true-negative rates. Since these rates were directly derived from the original data, correlations between test results in sequential strategies were incorporated into the outcome. False positive and false negative results were equally rated as incorrect classification.

#### Costs

Costs were assessed from a health care perspective. For each of the four tests, costs in Euros were calculated (Table 2). These costs were based on Dutch prices for test materials and costs of labour time of GP assistants, who generally perform urine investigations in Dutch primary care. For all tests except the dipslide, a labour time of one consultation with the GP assistant (10 minutes) was used. The result of a dipslide can be determined only after one day. Therefore, for the dipslide a labour time of 1.5 consultations was used. This was based on a poll we performed among 30 GPs, of whom 15 GPs charged one, and 15 charged two consultations when adding a dipslide to the diagnostic work up. Costs of antibiotic treatment were estimated to be €6.76 (5 days nitrofurantoin 100 mg– €1.06[14] and delivery costs—€5.70[15]). All diagnoses of UTI were assumed to be followed by a course of antibiotic treatment. All costs were estimated for the year 2011.

**Table 2:** Total costs for each test (history questions, dipstick, sediment and dipslide), subdivided into costs per test component.

Test	Material	Consultations	Total costs
History	None	1 consultation (€7.28)	€7.28
Dipstick	1 urine stick (€0.63)	1 consultation (€7.28)	€7.91
Sediment	1 microscope slide (€0.05)	1 consultation (€7.28)	€8.94
	Microscope (€1.11)		
	Centrifuge (€0.50)		
Dipslide	1 dipslide (€0.90)	1.5 consultations (€10.92)	€12.02
	Incubator (€0.20)		

### Cost-effectiveness analysis

#### Deterministic analysis

The expected costs and the expected proportion of correctly classified women per diagnostic strategy were estimated using the decision trees. For each end node in the tree, effects and costs were weighted by their probability of occurring. Subsequently, the strategies were ordered according to increasing costs and, if costs were equal, to increasing percentage of correctly classified women. The next step was to determine whether there were strategies that were dominated by other strategies (i.e. the latter were less expensive and more effective) or that were subject to extended dominance (i.e. dominated by a combination of other strategies).[16] Subsequently, among non-dominated strategies, incremental cost-effectiveness ratios (ICERs) were calculated between adjacent strategies starting with the least costly strategy. All strategies were plotted in a cost-effectiveness (CE) plane. In a CE plane, effects are plotted on the x-axis and costs on the y-axis. The non-dominated strategies together make up the efficiency frontier. The slope of this frontier indicates the additional monetary investments needed to gain one additional unit of effect; that is, one additional correctly classified woman.

#### Probabilistic analysis

In addition to the deterministic analysis, a probabilistic analysis was performed.[17] For the probabilistic analysis, beta distributions were fitted for the prevalence of UTI, and the number of diagnosed events and non-events per test conditional on other tests included in a strategy. Next, using Monte Carlo simulation techniques 1000 random draws were taken from these distributions. Uncertainty surrounding the proportion of accurately classified women was estimated using 95% credibility intervals (CrI) by estimating the 2.5% and 97.5% percentiles. Costs were considered to be fixed, and were, therefore, not varied. The results from the probabilistic analysis were used to estimate cost-effectiveness acceptability curves (CEACs) according to the net-benefit framework.[18] A CEAC shows, for a range of different ceiling ratios, the probability that a strategy is cost-effective. A ceiling ratio represents the additional amount of money that society would be willing to pay per additional correctly classified woman; these additional costs are on top of the costs of the reference strategy. The ceiling ratios are plotted on the x-axis, and the probability that a strategy is considered cost-effective on the y-axis.[19]

#### Univariate sensitivity analysis

In practice, not all clinicians have both a sediment and a dipslide at their disposal, depending on, for example, individual preferences, local availability and national guidelines. Therefore, in a first sensitivity analysis, all strategies containing a dipslide were excluded and in a second one, all strategies containing a sediment. In addition, in a third sensitivity analysis, the effect of different prevalence rates on the efficiency frontier was evaluated. In this analysis, the prevalence of UTI was varied by using estimates that were 10% and 20% higher and lower than the observed prevalence of 61%. Finally, to be able to show that the most cost-effective strategies improve patient outcomes as compared to no testing or treating all patients, we conducted a sensititivy analyses in which two additional treatment strategies were added. First, a treat-none strategy in which none of the women received UTI treatment and second, a treat-all strategy in which all women received UTI treatment without further testing.

## Results

Data from 196 women who contacted their GP with painful and/or frequent micturation were used in the cohort study. Of these women, 120 (61%) had a UTI based on a positive culture (≥10^3^ CFU/ml).

### Diagnostic tests

Table 1 shows the 15 analyzed strategies with their positive and negative predictive values as well as their sensitivities and specificities. PPVs were at least 0.75 for all strategies; NPVs increased when more sequential or parallel tests were performed (with the exception of strategy 13, which was the only strategy in which a subsequent test was performed if the previous test result was positive).

### Cost-effectiveness

#### Deterministic analysis

Estimates of the expected proportions of correctly classified women and expected costs for all strategies are presented in Table 3. The strategy in which a positive history was followed up with a dipstick (strategy 13) had the lowest costs (€9.13) but also resulted in a relatively low proportion of correctly classified women (0.59). Performing both a history and dipstick (strategy 7) increased the proportion of correctly classified women considerably to 0.73 while costs only marginally increased (€10.53). Sequentially adding either a sediment or a dipslide after a negative history and dipstick (strategies 3 and 4, respectively) increased the proportion of correctly classified women (0.87 and 0.88, respectively), but at the expense of higher costs (€12.72 and €13.74, respectively). Performing two or more tests simultaneously (strategies 8–10, 12) resulted in lower proportions of correctly classified women compared to the strategies consisting of sequential tests, while costs increased considerably.

**Table 3:** Expected proportion of correctly classified women, expected costs and incremental cost per correctly classified woman for all test strategies in the main analysis and the two univariate sensitivity analyses excluding strategies containing a dipslide and strategies containing a sediment.

					Main analysis	Without dipslide	Without sediment
Strategy		Expected proportion correctly classified women (95% CrI)	UTI positives/true positives	Expected cost (95% CrI)	Incremental cost per correctly classified woman	Incremental cost per correctly classified woman	Incremental cost per correctly classified woman
13	History +, Dipstick	0.59 (0.52; 0.65)	0.24/0.22	9.13 (8.72; 9.57)	Reference	Reference	Reference
1	History	0.65 (0.58; 0.71)	0.41/0.34	10.08 (9.63; 10.54)	Dominated	Dominated	Dominated
6	Dipstick	0.68 (0.61; 0.75)	0.32/0.30	10.09 (9.65; 10.55)	Dominated	Dominated	Dominated
7	History & Dipstick	0.73 (0.67; 0.79)	0.39/0.37	10.53 (10.08; 10.95	9.37	9.37	9.37
2	History -, Dipstick	0.79 (0.73; 0.84)	0.57/0.49	11.48 (11.06; 11.90)	Dominated	Dominated	16.70
14	Dipstick -, History	0.76 (0.70; 0.82)	0.55/0.46	11.63 (11.15; 12.08)	Dominated	Dominated	Dominated
3	History -, Dipstick -, Sediment	0.87 (0.82; 0.91)	0.68/0.58	12.72 (12.43; 13.01)	15.89	15.96	Excluded
8	History & Dipstick & Sediment	0.81 (0.75; 0.86)	0.51/0.47	13.01 (12.51; 13.50)	Dominated	Dominated	Excluded
4	History -, Dipstick -, Dipslide	0.88 (0.83; 0.92)	0.67/0.58	13.74 (13.61; 13.88)	102.94	Excluded	25.00
15	Dipstick -, Dipslide	0.81 (0.74; 0.86)	0.48/0.45	14.36 (14.01; 14.71)	Dominated	Excluded	Dominated
5	History -, Dipstick -, Sediment -, Dipslide	0.88 (0.83; 0.92)	0.69/0.59	14.38 (14.17; 14.62)	Dominated	Excluded	Excluded
11b	Dipslide cut-off 105 CFU/ml	0.78 (0.72; 0.83)	0.46/0.43	15.14 (14.67; 15.61)	Dominated	Excluded	Dominated
9	History & Dipstick & Dipslide	0.81 (0.76; 0.86)	0.45/0.44	15.71 (15.24; 16.23)	Dominated	Excluded	Dominated
12	Dipstick & Dipslide	0.82 (0.76; 0.87)	0.46/0.44	15.75 (15.28; 16.24)		Excluded	Dominated
11a	Dipslide cut-off 103 CFU/ml	0.73 (0.66; 0.79)	0.68/0.51	16.61 (16.13; 17.05)		Excluded	Dominated
10	History & Dipstick & Sediment & Dipslide	0.83 (0.78; 0.88)	0.48/0.46	17.58 (17.10; 18.07)		Excluded	Excluded

95% CrI = 95% Credibility Interval

For the dipslide, a cut-off value of ≥10^5^ CFU/mL was used in all strategies, except for strategy 11a. This strategy consisted of the dipslide as a single test at a cut-off value of ≥10^3^ CFU/mL. The strategies are ordered according to increasing costs, and, if costs are equal, increasing percentage of correctly classified women. For dominating strategies (= more accurate than any single strategy or combination of strategies that is equally or less costly), ICERs were calculated.

**Fig 1:**
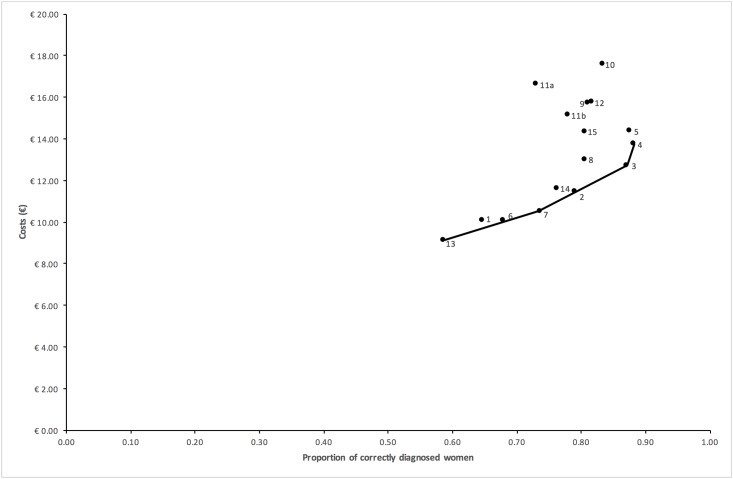
Cost-effectiveness plane. The cost-effectiveness plane shows for each strategy the expected proportion of correctly classified women (x-axis) and expected costs (y-axis). By drawing a line between strategies 13, 7, 3 and 4 (dominant strategies) the efficiency frontier is revealed.

In columns 3, 4 and 5 of Table 3, the incremental cost-effectiveness ratios are shown. Based on the principles of (extended) dominance, 12 strategies (strategies 1, 2, 5, 6, 8, 9, 10 11a, 11b, 12, 14, and 15) were excluded from the comparison. This resulted in an efficiency frontier consisting of strategies 13 (dipstick if history was positive), 7 (history and dipstick), 3 (sediment if history and subsequent stick were negative) and 4 (dipstick if history was negative, followed by dipslide if dipstick was negative), as is displayed graphically in the CE plane in Fig 1.

#### Probabilistic analysis

**Fig 2:**
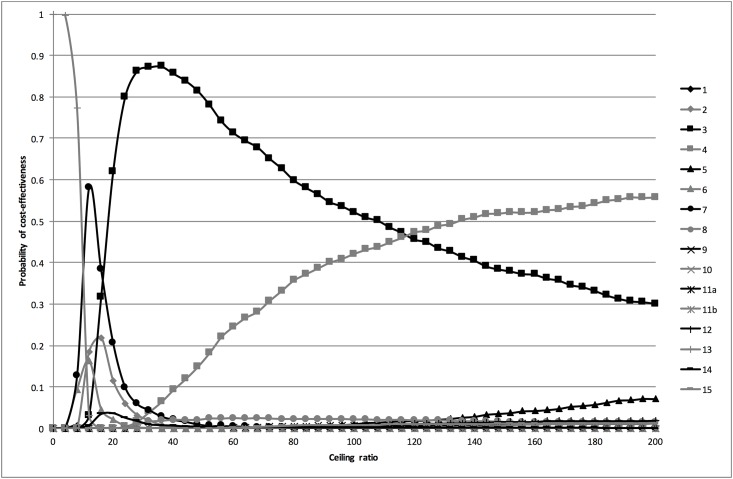
Cost-effectiveness acceptability curves for the main analysis. The cost-effectiveness acceptability curves are based on the probabilistic analysis. They show the probability that the different diagnostic strategies are cost-effective at a range of ceiling ratios.

The probabilistic analysis revealed a fair amount of uncertainty around the expected percentages of correctly classified women as shown by the 95% CrI in Table 3. The CEACs in Fig 2 show that for ceiling ratios below €10 per additional correctly classified woman, strategy 13 (dipstick if history was positive) had the highest probability of being cost-effective. For ceiling ratios between €10 and €17 per additional correctly classified woman, this was strategy 7 (history and dipstick); for ceiling ratios between €17 and €118 per additional correctly classified woman strategy 3 (sediment if history and subsequent stick were negative); and for ceiling ratios of more than €118 per additional correctly classified woman strategy 4 (dipstick if history was negative, followed by dipslide if dipstick was negative).

#### Univariate sensitivity analyses

**Fig 3:**
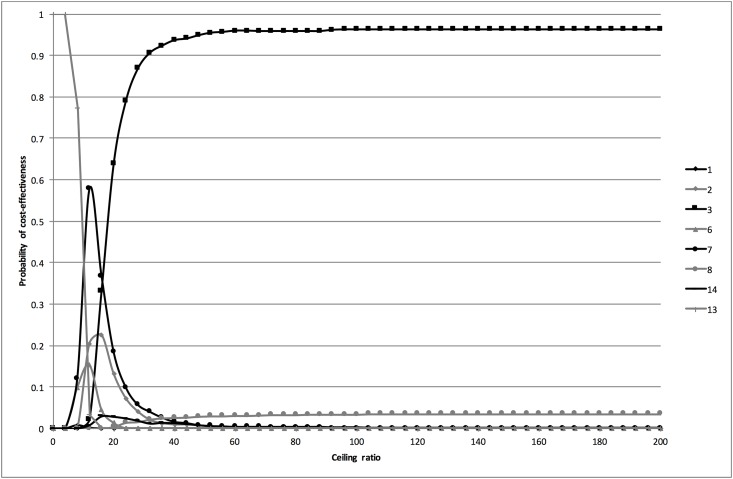
Cost-effectiveness acceptability curves for the analysis excluding the dipslide. The cost-effectiveness acceptability curves are based on the probabilistic analysis. They show the probability that the different diagnostic strategies are cost-effective at a range of ceiling ratios.

The results of the univariate sensitivity analyses excluding the dipslide and sediment are presented in Table 3. In the sensitivity analysis excluding strategies containing a dipslide, the efficiency frontier consisted of strategies 13 (dipstick if history was positive), 7 (history & dipstick) and 3 (sediment if history and subsequent stick were negative). The corresponding CEACs in Fig 3 show that for ceiling ratios below €10 per additional correctly classified woman strategy 13 was most likely to be cost-effective, for ceiling ratios between €10 and €16 strategy 7, and for ceiling ratios of more than €16 strategy 3.

**Fig 4:**
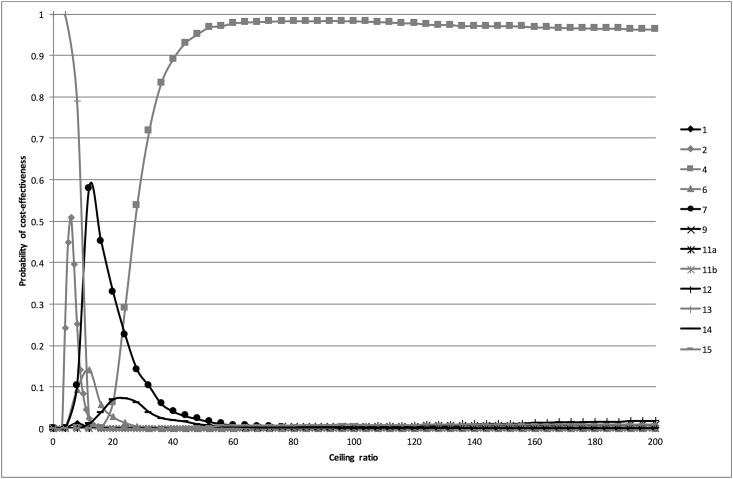
Cost-effectiveness acceptability curves for the analysis excluding the sediment. The cost-effectiveness acceptability curves are based on the probabilistic analysis. They show the probability that the different diagnostic strategies are cost-effective at a range of ceiling ratios.

Exclusion of strategies containing a sediment resulted in an efficiency frontier consisting of strategies 13 (dipstick if history was positive), 7 (history & dipstick), and 4 (dipslide if history and subsequent stick were negative). The CEACs in Fig 4 show that for ceiling ratios below €10 per additional correctly classified woman strategy 13 was most the cost-effective strategy, for ceiling ratios between €10 and €26 strategy 7, and for ceiling ratios of more than €26 strategy 4.

**Fig 5:**
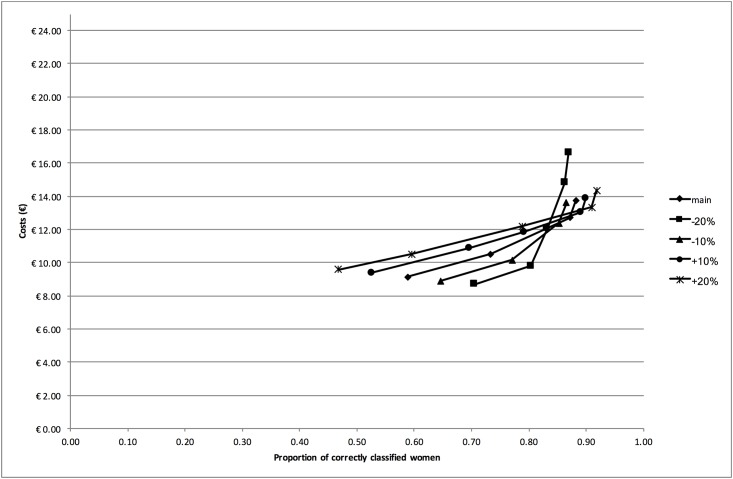
Cost-effectiveness plane for the univariate sensitivity analysis. The cost-effectiveness plane shows the efficiency frontiers for the univariate sensitivity analyses in which the prevalence of urinary tract infection was varied by plus and minus 10% and 20%.

Fig 5 shows the efficiency frontiers for the sensitivity analysis in which the observed prevalence (61%) was varied by plus and minus 10% and 20%. When the prevalence is 20% lower than the observed strategy, strategy 4 (dipslide if history and subsequent stick were negative) was replaced by strategies 12 (dipstick & dipslide) and 10 (history & dipstick & sediment & dipslide) on the efficiency frontier. When the prevalence of UTI was decreased with 10%, the efficiency frontier remained the same as in the main analysis. However, when the prevalence was increased with 10%, strategy 2 (dipstick if history was negative) became the dominant strategy after strategies 13 (dipstick if history was positive) and 7 (history & dipstick) and was followed by strategies 3 (sediment if history and subsequent stick were negative) and 4 (dipslide if history and subsequent stick were negative). When the prevalence is 20% higher than the observed prevalence, strategy 1 (history only) replaced strategy 7 (history & dipstick) on the efficiency frontier and strategy 5 (dipstick if history was negative, followed by sediment if dipstick was negative, followed by dipslide if sediment was negative) replaced strategy 4 (dipslide if history and subsequent stick were negative).

**Fig 6:**
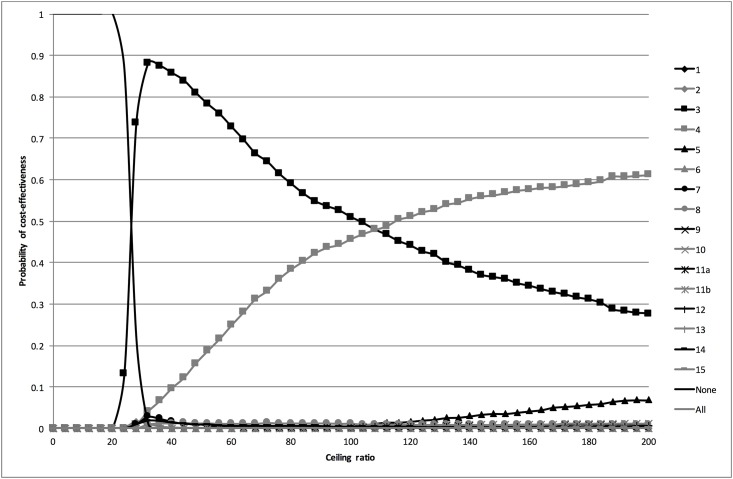
Cost-effectiveness acceptability curves for the analysis including treat-none and treat-all strategies. The cost-effectiveness acceptability curves are based on the probabilistic analysis. They show the probability that the different diagnostic strategies are cost-effective at a range of ceiling ratios.

Inclusion the treat-none and treat-all strategies resulted in replacement of strategies 13 (dipstick if history was positive) and 7 (history and dipstick) by the treat-none strategy (see Fig 6). In this sensitivity analysis, for ceiling ratios between €0 and €26 per additional correctly classified woman the treat-none strategy had the highest probability of being cost-effective; for ceiling ratios between €27 and €108 per additional correctly classified woman strategy 3 (sediment if history and subsequent stick were negative); and for ceiling ratios of more than €108 per additional correctly classified woman strategy 4 (dipstick if history was negative, followed by dipslide if dipstick was negative).

## Discussion

A decision-analytic model based on decision trees was developed to compare the cost-effectiveness of 15 different strategies for diagnosing a UTI in women who contact their general practitioner (GP) with painful and/or frequent micturition. Most sequential testing strategies resulted in higher proportions of correctly classified women and lower costs than parallel testing strategies. Results from the probabilistic sensitivity analysis showed that the strategy of choice depends on society’s willingness to pay for one additional correctly classified woman (ceiling ratio). If the ceiling ratio is less than €10, the most cost-effective diagnostic strategy is to perform a dipstick if history is positive. At a ceiling ratio between €10 and €17, the most cost-effective strategy is to combine history and dipstick. For ceiling ratios between €17 and €118, the most cost-effective strategy is to perform a sediment if history and subsequent stick are negative. For ceiling ratios of more than €118 or more, the most cost-effective strategy is to perform a dipslide if history, subsequent stick and (in case of a negative stick result) subsequent sediment are negative. Sensitivity analyses showed that a treat-none strategy had the highest probability of being cost-effective for ceiling ratios until €26 per additional correctly classified woman. For higher ceiling ratios, results did not change as compared to the main analysis. The results of the sensitivity analyses in which the prevalence of UTI was varied showed that at a 20% lower prevalence, parallel testing strageties become most cost-effective at higher ceiling ratios. At lower prevalences, healthcare providers are probably more reluctant to treat patients with antibiotics making the specificity of the diagnostic strategy more important than the sensitivity. At higher prevalences, however, healthcare providers are more willing to start antibiotics treatment and, thus, are more interested in correctly identifying women with a UTI as such (i.e. sensitivity).

Although ceiling ratios exist for quality-adjusted life-years gained, such willingness-to-pay values are not available for one additional woman correctly diagnosed with a UTI. Thus, definition of such a ceiling ratio is not straight-forward. Incorrect classification of a woman presenting with UTI symptoms may result in additional GP consultations (€29 per consultation) or absenteeism days (€215 per day). Assuming that the ceiling ratio lies between these values, would mean that a strategy in which a sediment is performed if history and subsequent stick are negative is the most cost-effective strategy. This is also the case when a treat-none strategy is taken into account.

In a previously published paper that used the same dataset, but focusses on the diagnostic value of the different tests, it was concluded that the sediment and dipslide have little added value.[12] However, our results show that performing a dipslide or sediment after a negative history and/or dipstick increases the proportion of correctly classified women considerably at moderate costs. This difference is explained by the fact that in the previously published paper the tests were simultaneously performed and a diagnosis of UTI was considered present if the patient had a predicted UTI risk of 70% or more. In this paper, a larger number of strategies were evaluated that consisted of both sequential and parallel testing. The present paper shows that when performing sequential tests for UTI, the dipslide and sediment do add value to the diagnostic process.

Our study has several strengths. First, the data on diagnostic accuracy were obtained from a cohort study in which all participating women underwent all available tests, including the reference standard (urine culture). This allowed for calculation of conditional sensitivities and specificities in all strategies, which is superior to pooling estimates from meta-analyses of single test studies.[20] Second, we used probabilistic sensitivity analysis to determine the probability for each diagnostic strategy to be cost-effective given different ceiling ratios. This is considered state-of-the-art methodology and takes into account the uncertainty in all included model parameters.[17] Third, the proportion of both correct positive and negative diagnoses was chosen as the primary outcome measure in this study. The advantage of using this outcome measure is that the overall performance of the specific strategy is considered. However, the number of false positive and negative diagnoses is not taken into account in this outcome measure. Although, the consequences of a false negative diagnosis may be limited (risk of severe complications of an UTI is low), a false positive diagnosis leads to unnecessary treatment with antibiotics of which the consequences are indirectly incorporated in the cost estimates in this study. However, unnecessary antibiotic treatment may also have adverse effects for the patient such as side effects of the treatment and for society because of an increased risk of antibiotic resistance. The proportions of positive and true positive diagnoses show that the proportion of unnecessary antibiotic treatments (proportion of false positive diagnoses) varies greatly between strategies from 0.01 (strategy 9) to 0.10 (strategies 3 and 5). Another potential disadvantage of this combined proportion of correctly classified women is that false negatives are not accounted for, that is there is no penalty for missing a diagnosis of UTI. False positives are indirectly incorporated in the outcomes, because in these cases costs for antibiotic treatment are included in the total costs per strategy which is a commonly used approach in cost-effectiveness analyses.

Our study also has some limitations. First, we did not incorporate the possible discomfort of the diagnostic delay of the dipslide into our analysis. Since the result of the dipslide is only known after one day, it might be less attractive to use in practice. Second, we did not take into account productivity losses due to absence from paid work because of UTI complaints. However, of all 196 participants in the cohort study, 171 (87%) had not missed any days at work due to their UTI symptoms. Since previous research has shown that productivity losses were relevant in only a minority of cases [21], we assume that productivity losses in this patient population were small. Third, an uncommon but costly complication of undiagnosed UTIs is pyelonephritis, which is associated with considerable costs. However, placebo arms of randomized trials suggest that UTIs in uncomplicated patients seldom progress to pyelonephritis.[22,23,24] In our study population, none of the participants developed pyelonephritis. Fourth, it might be questioned whether other strategies would be considered cost-effective if the UTI prevalence changed. However, the UTI prevalence in our study (61%) was similar to previously described prevalences in primary care, suggesting that our results can be applied to other primary care settings.[11,25,26,27] In addition, uncertainty surrounding the prevalence of UTI in women presenting to their GP with painful and/or frequent micturation was addressed in both a probabilistic analysis and in a univariate sensitivity analysis in which the prevalence was varied. Fifth, in our study, to reduce inter-observer variation and improve interpretability, sediment investigations were performed in a laboratory by trained laboratory technicians. In general practice, however, the circumstances are seldom optimal, which may negatively affect the diagnostic properties of the sediment resulting in either over- or under diagnosis.[28]

It should be noted that although costs of antimicrobial resistance are relevant for clinical decision making in UTI management on the long-term, we did not take this factor into account in our analyses for two reasons. First, since resistance rates differ substantially between antibiotics and between regions),[2,3,29,30], it would be difficult to develop a generally applicable model. Moreover, the data were obtained in a setting in which the rate of multidrug resistant bacteria is low (Dutch general practice). Second, the majority of costs associated with antimicrobial resistance are not related to the use of expensive antibiotics for this specific patient, but with the possible cost savings associated with prudent antibiotic use in the long term.

UTI diagnosis in primary care is generally based on negative triage: if a test result is negative, additional tests are administered; if a test result is positive, a UTI is considered to be present and no more tests are done. Therefore, we based all sequential strategies on negative triage, with the exception of strategy 13. However, this strategy was not part of the efficiency frontier, and was, therefore, not considered cost-effective in comparison with negative triage strategies.

In conclusion, depending on decision makers’ willingness to pay for one additional correctly classified woman, the strategy consisting of performing a history and dipstick simultaneously (ceiling ratios between €10 and €17) or performing a sediment if history and subsequent dipstick are negative (ceiling ratios between €17 and €118) are the most cost-effective strategies to diagnose a UTI.

## Supporting information

S1 Appendix Decision trees. For each strategy, a tree is shown that depicts the test sequence and all possible test results. The end nodes of the trees summarize the diagnostic conclusions (false positive, true positive, true negative, false negative) and the total costs of the preceding branches.
(PDF)
