# Supplementary material for: Cost-effectiveness of different strategies for diagnosis of uncomplicated urinary tract infections in women presenting in primary care
Source: PLoS One. 2017 Nov 29;12(11):e0188818. doi: 10.1371/journal.pone.0188818 (PMC5706710; doi:10.1371/journal.pone.0188818)
Supplement: S1 Appendix — For each strategy, a tree is shown that depicts the test sequence and all possible test results. The end nodes of the trees summarize the diagnostic conclusions (false positive, true positive, true negative, false negative) and the total costs of the preceding branches. (PDF) [file pone.0188818.s001.pdf]

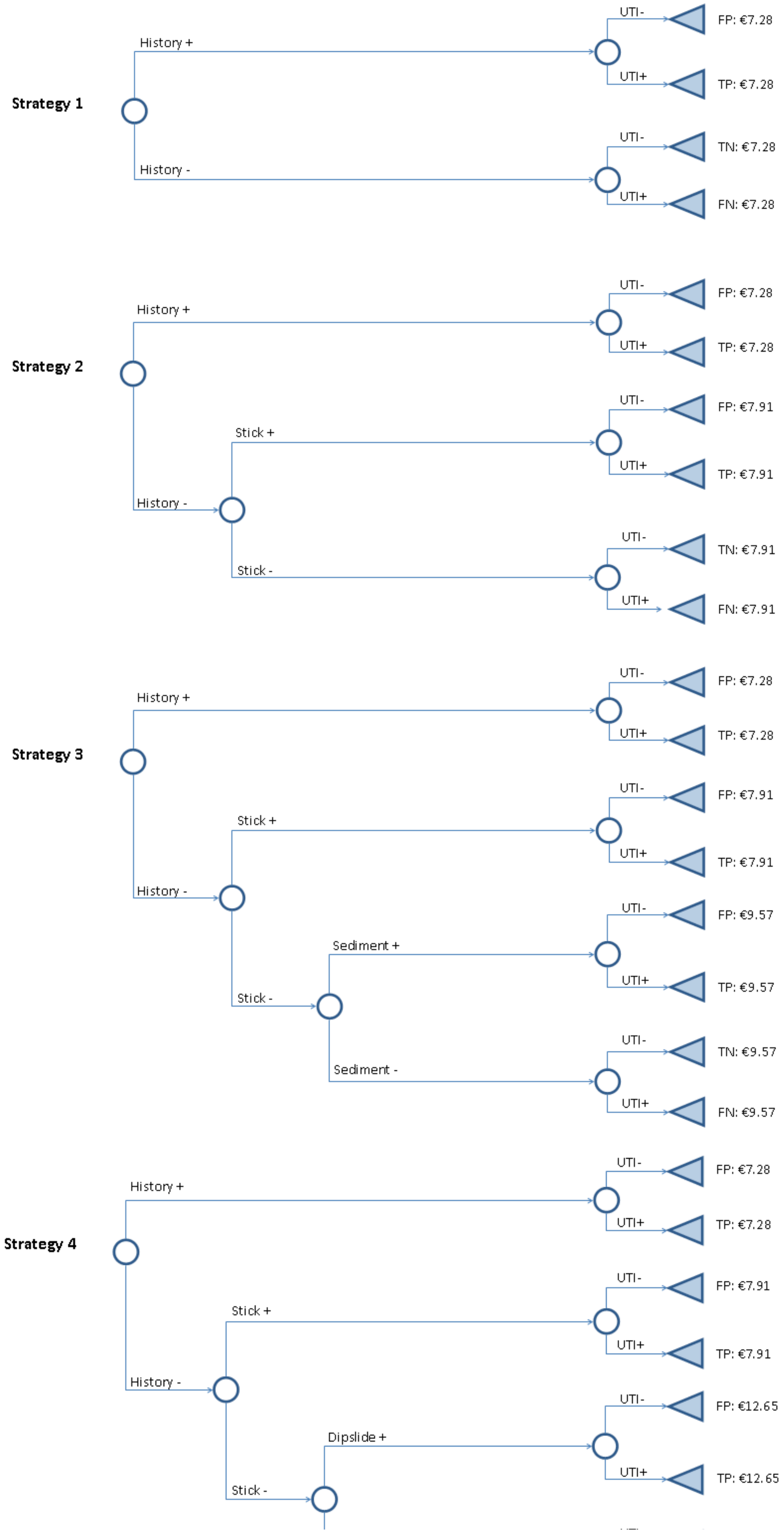
Strategy 1
History +
History -
UTI-
UTI+
FP: €7.28
TP: €7.28
TN: €7.28
FN: €7.28
Strategy 2
History +
History -
Stick +
Stick -
FP: €7.28
TP: €7.28
FP: €7.91
TP: €7.91
TN: €7.91
FN: €7.91
Strategy 3
History +
History -
Stick +
Stick -
Sediment +
Sediment -
FP: €7.28
TP: €7.28
FP: €7.91
TP: €7.91
FP: €9.57
TP: €9.57
TN: €9.57
FN: €9.57
Strategy 4
History +
History -
Stick +
Stick -
Dipslide +
FP: €7.28
TP: €7.28
FP: €7.91
TP: €7.91
FP: €12.65
TP: €12.65

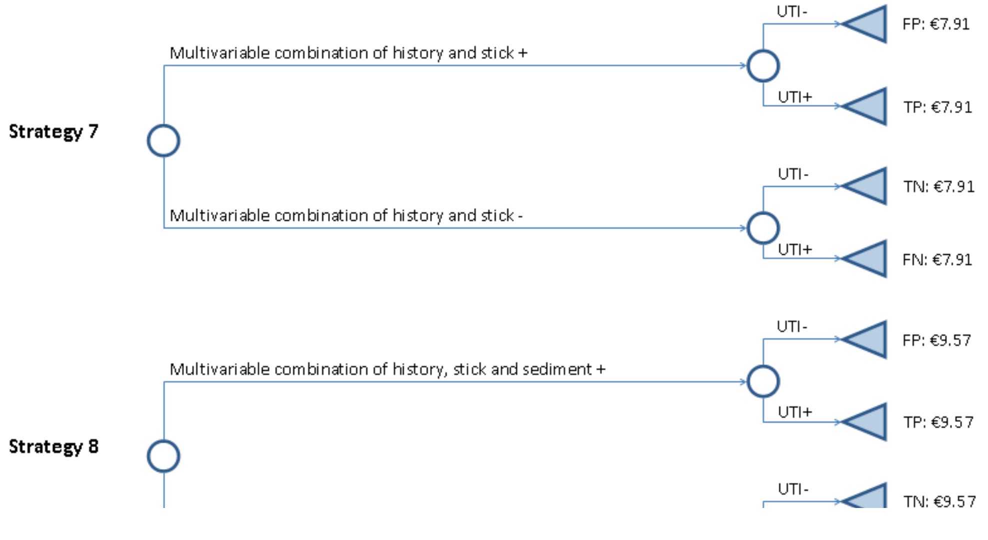
Strategy 7
Multivariable combination of history and stick +
UTI-
FP: €7.91
UTI+
TP: €7.91
Multivariable combination of history and stick -
UTI-
TN: €7.91
UTI+
FN: €7.91
Strategy 8
Multivariable combination of history, stick and sediment +
UTI-
FP: €9.57
UTI+
TP: €9.57
UTI-
TN: €9.57

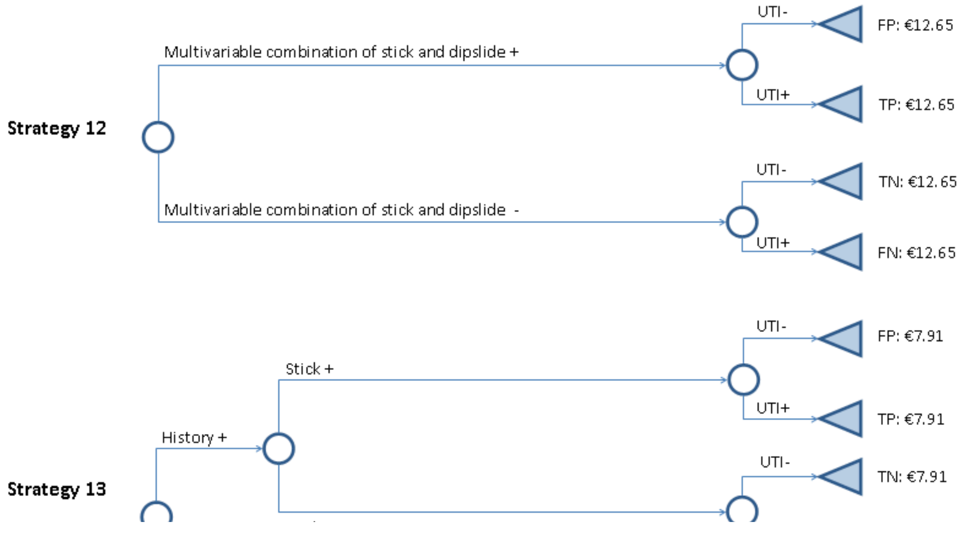
Strategy 12
Multivariable combination of stick and dipslide +
UTI-
FP: €12.65
UTI+
TP: €12.65
Multivariable combination of stick and dipslide -
UTI-
TN: €12.65
UTI+
FN: €12.65
Strategy 13
History +
Stick +
UTI-
FP: €7.91
UTI+
TP: €7.91
UTI-
TN: €7.91

* For the dipslide, a cut-off value of $\geq 10^5$ CFU/mL was used in all strategies, except for strategy 11a. This strategy consisted of the dipslide as a single test at a cut-off value of $\geq 10^3$ CFU/mL.
